# Supplementary figures and images for: Genetic evidence for functional diversification of gram-negative intermembrane phospholipid transporters
Source: PLoS Genet. 2024 Jun 24;20(6):e1011335. doi: 10.1371/journal.pgen.1011335 (PMC11226057; doi:10.1371/journal.pgen.1011335)

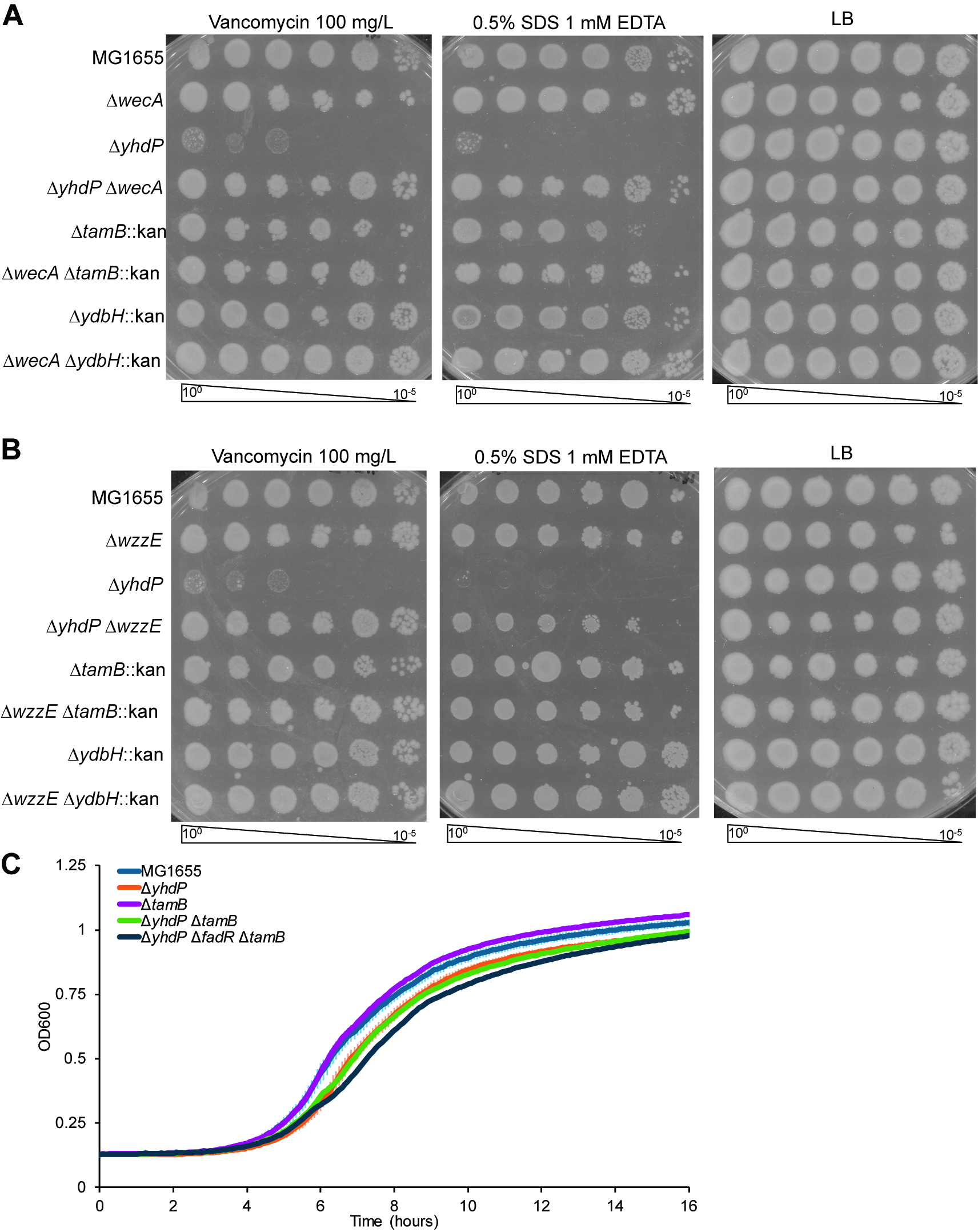

Supplement: S1 Fig — (A-B) Efficiency of plating assays (EOPs) were performed at 37°C in the indicated conditions. Only ΔyhdP causes significant outer membrane (OM) permeability to vancomycin and SDS (sodium dodecyl sulfate) EDTA. Loss of all enterobacterial common antigen due to ΔwecA deletion (A) or loss of cyclic enterobacterial common antigen due to ΔwzzE (B) suppresses ΔyhdP OM permeability but does not change ΔtamB or ΔydbH phenotypes. Data are representative of three independent experiments. (TIF) [file pgen.1011335.s001.tif]

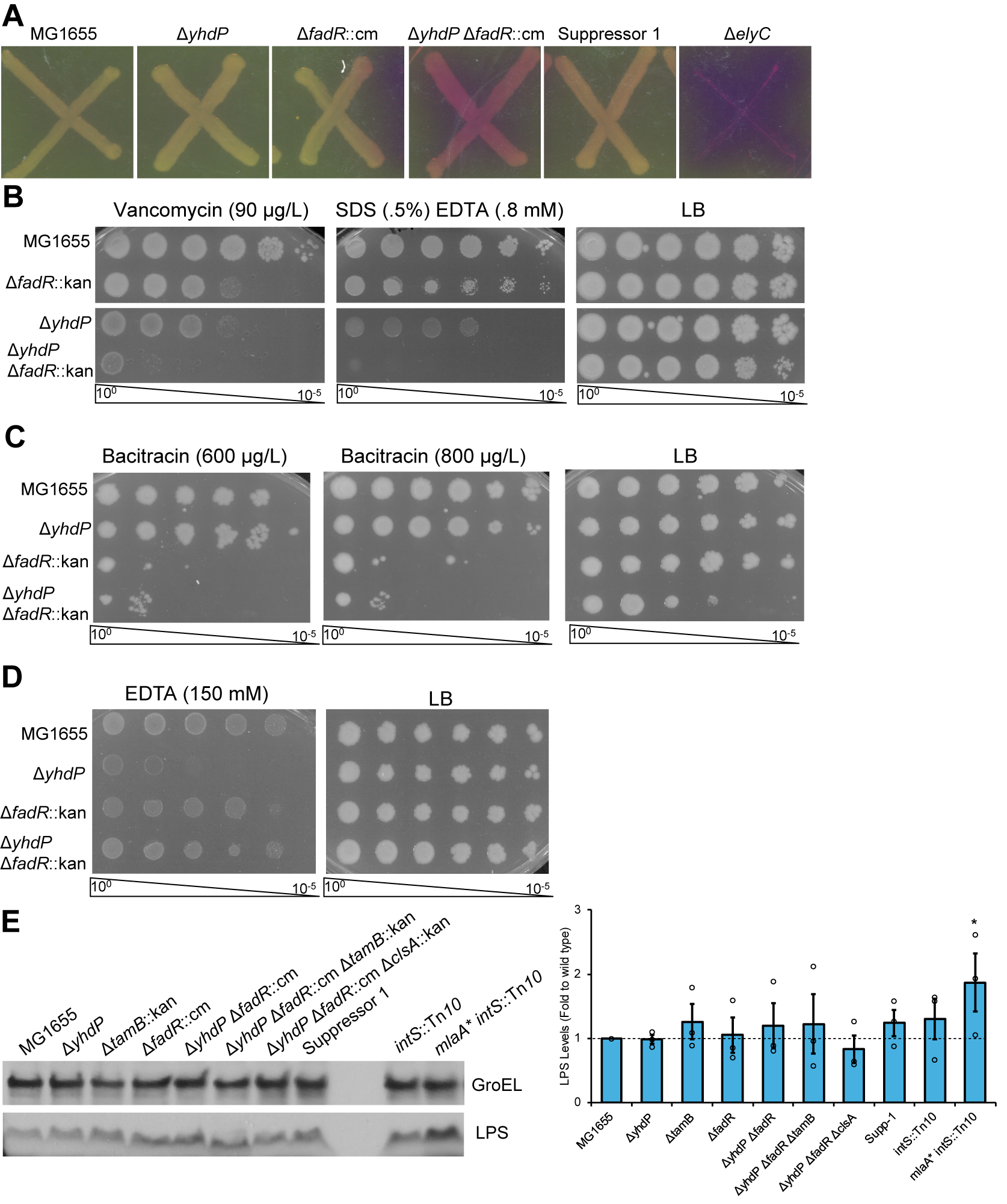

Supplement: S2 Fig — (A) A CPRG assay was used to assay lysis and envelope permeability. Red color indicates the production of chlorophenol red after β-galactosidase cleavage of CPRG. The ΔyhdP ΔfadR strain shows increased lysis or envelope permeability compared to the wild type strain and single mutants. The ΔelyC strain serves as a positive control. (B-D) Sensitivity of strains to various compounds inhibited by the OM was assayed by EOP at 37°C. (B) The ΔyhdP ΔfadR strain shows additive sensitivity to vancomycin and SDS EDTA when compared to its parent strains. (C) The ΔyhdP ΔfadR strain demonstrates similar bacitracin sensitivity to the ΔfadR strain. (D) The ΔyhdP ΔfadR strain does not exhibit the EDTA sensitivity of a ΔyhdP strain. (E) LPS levels in cultures grown to OD = 0.2 at 37°C then down shifted to 30°C for two hours were assayed by immunoblot analysis (α-LPS core, Hycult Biotechnology) as has been described [30,43,105]. GroEL (Millipore Sigma) serves as a loading control. The mlaA* strain has increased LPS levels and serves as a control. Relative LPS levels from three biological replicates were determined by densitometry and are shown as the mean ± the SEM with individual data points. The mlaA* strain has the only significant change in LPS levels. All images are representative of three independent experiments. (TIF) [file pgen.1011335.s002.tif]

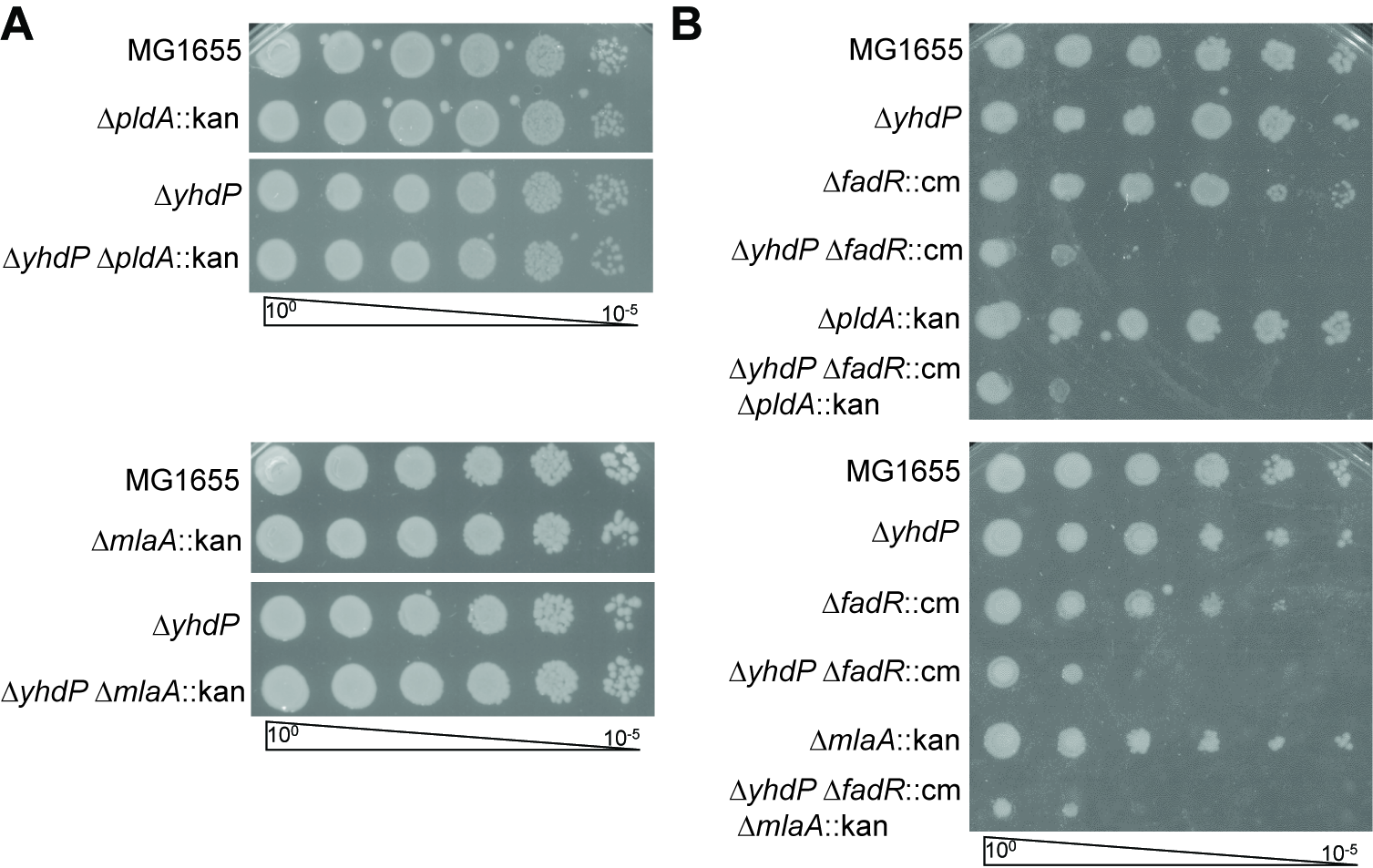

Supplement: S3 Fig — EOPs were carried out at 30°C on LB media. (A) Combination of ΔyhdP with ΔpldA or ΔmlaA did not result in cold sensitivity. (B) Combination of ΔyhdP ΔfadR with ΔpldA or ΔmlaA did not suppress cold sensitivity. Images are representative of three independent experiments. (TIF) [file pgen.1011335.s003.tif]

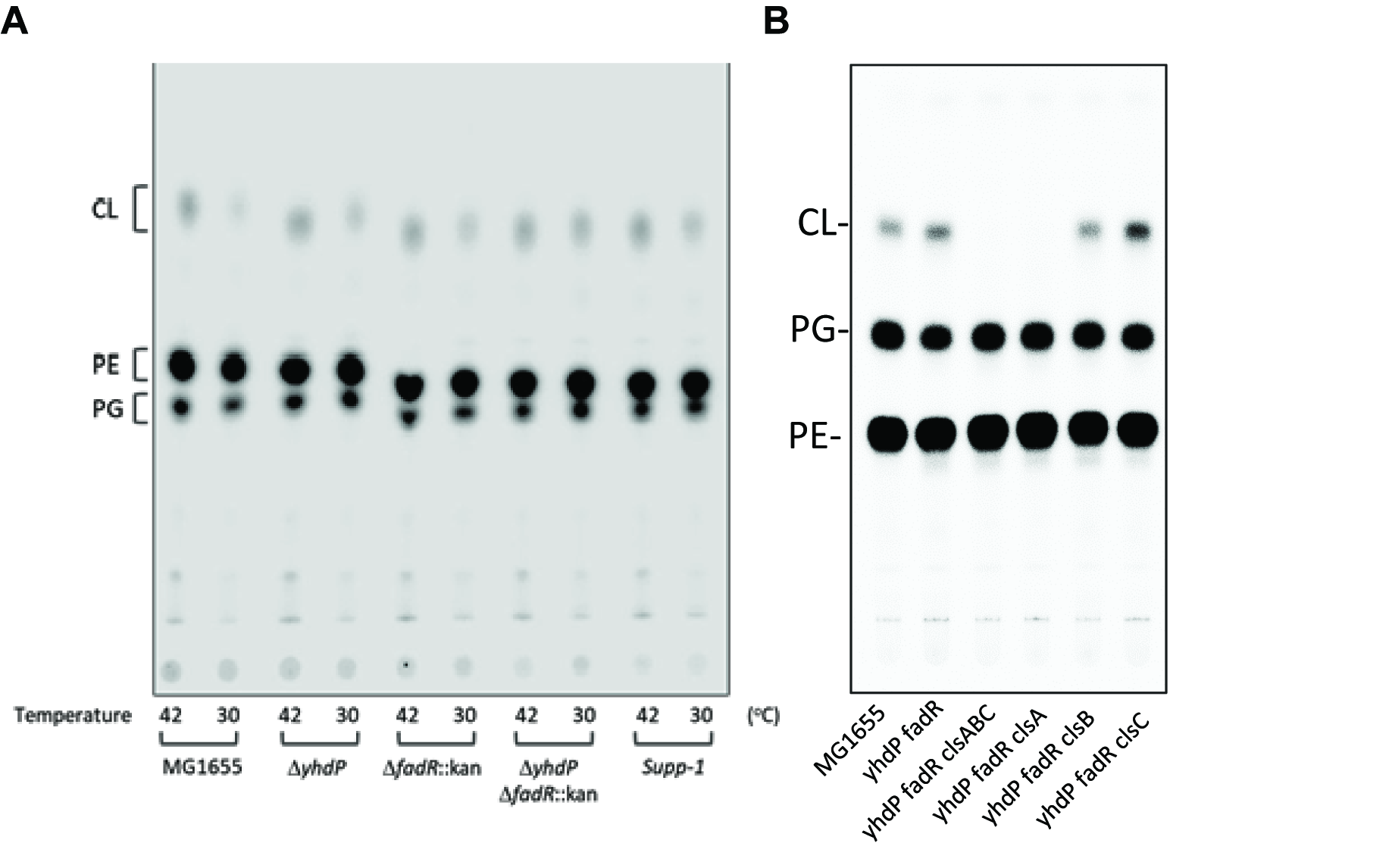

Supplement: S4 Fig — Cultures of the indicated strains were grown to log phase at 42°C then transferred to 30°C or 42°C for 2 hours before performing thin layer chromatography to analyze phospholipid content. Two representative images are shown. Solvent conditions are different between image (A) (chloroform-methanol-acetic acid [60/25/10] (vol/vol/vol)) and (B) (chloroform-methanol-ammonia-water [65/37.5/3/1] (vol/vol/vol/vol)) to allow better separation of PG and PE. CL: cardiolipin; PE: phosphatidylethanolamine; PG: phosphatidylglycerol. (TIF) [file pgen.1011335.s004.tif]

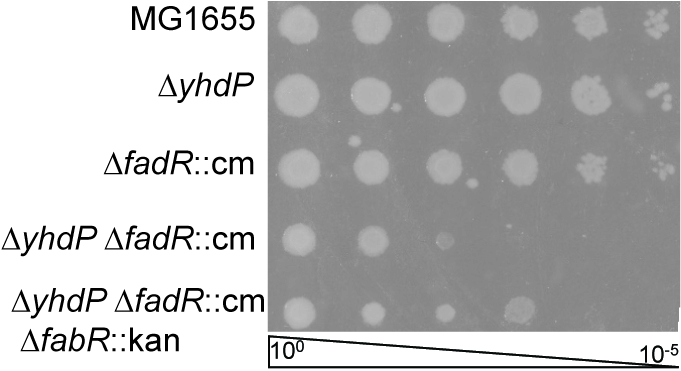

Supplement: S5 Fig — EOPs were performed at 30°C on LB media with the indicated strains. Deletion of fabR partially suppresses the cold sensitivity of the ΔyhdP ΔfadR strain. Image is representative of three independent experiments. (TIF) [file pgen.1011335.s005.tif]

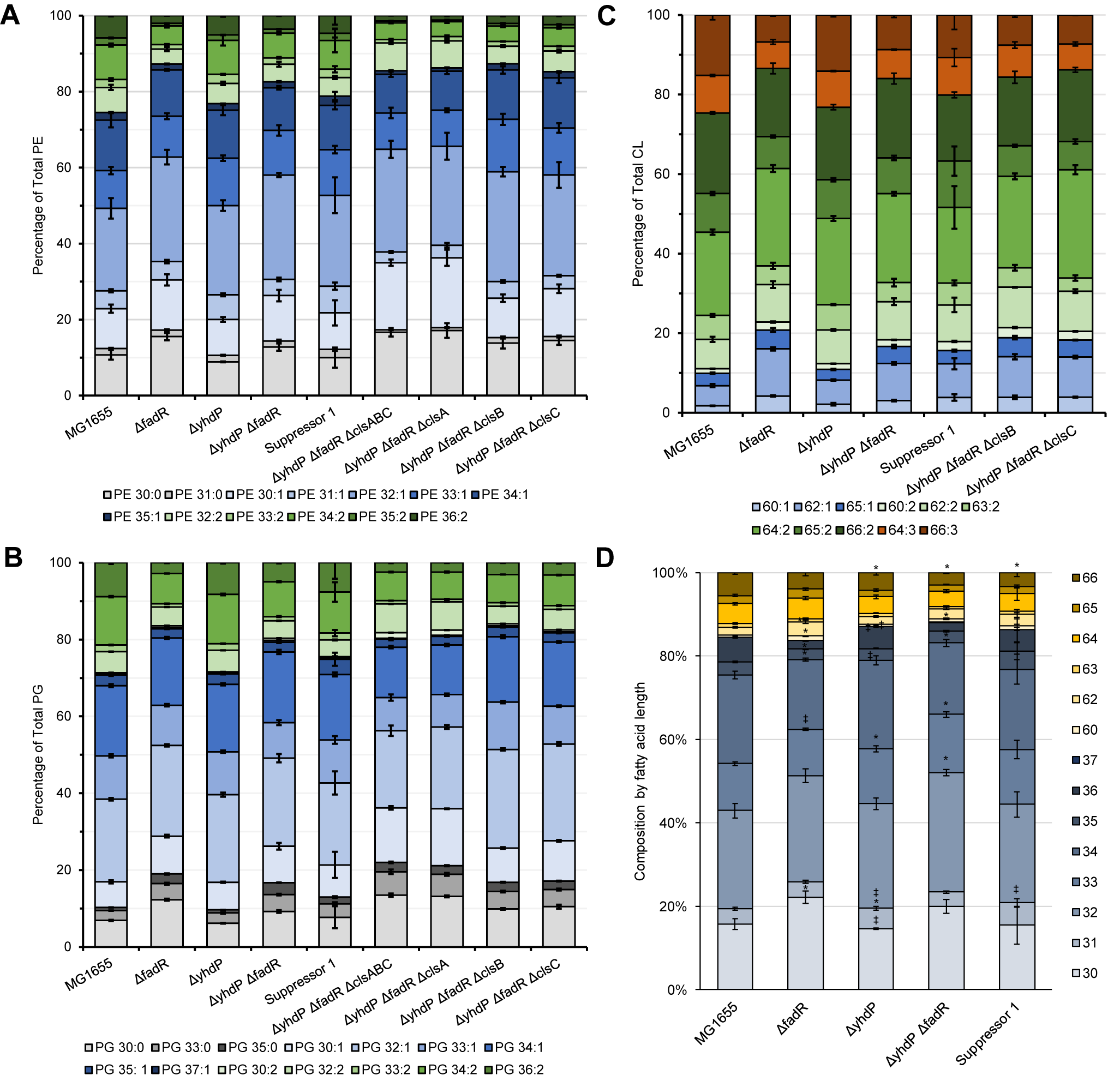

Supplement: S6 Fig — Phospholipid composition of the indicted strains was assayed using LC/MS after growth to OD600 = 0.2 at 42°C then down shifting the temperature to 30°C for 2 hours. (A-C) Percentages of PE (A), PG (B), and CL (C) were calculated from absolute quantification of all species detected. Data for phospholipids without unsaturations are shown in grey, with one unsaturation in blue, with two unsaturations in green, with three unsaturations in orange. (D) Total fatty acid lengths for all phospholipids are shown with CL specific lengths shown in yellow tones. * p<0.05 vs. MG1655 by the Mann-Whitney test; ‡ p<0.05 vs. the ΔyhdP ΔfadR strain by the Mann-Whitney test. (TIF) [file pgen.1011335.s006.tif]

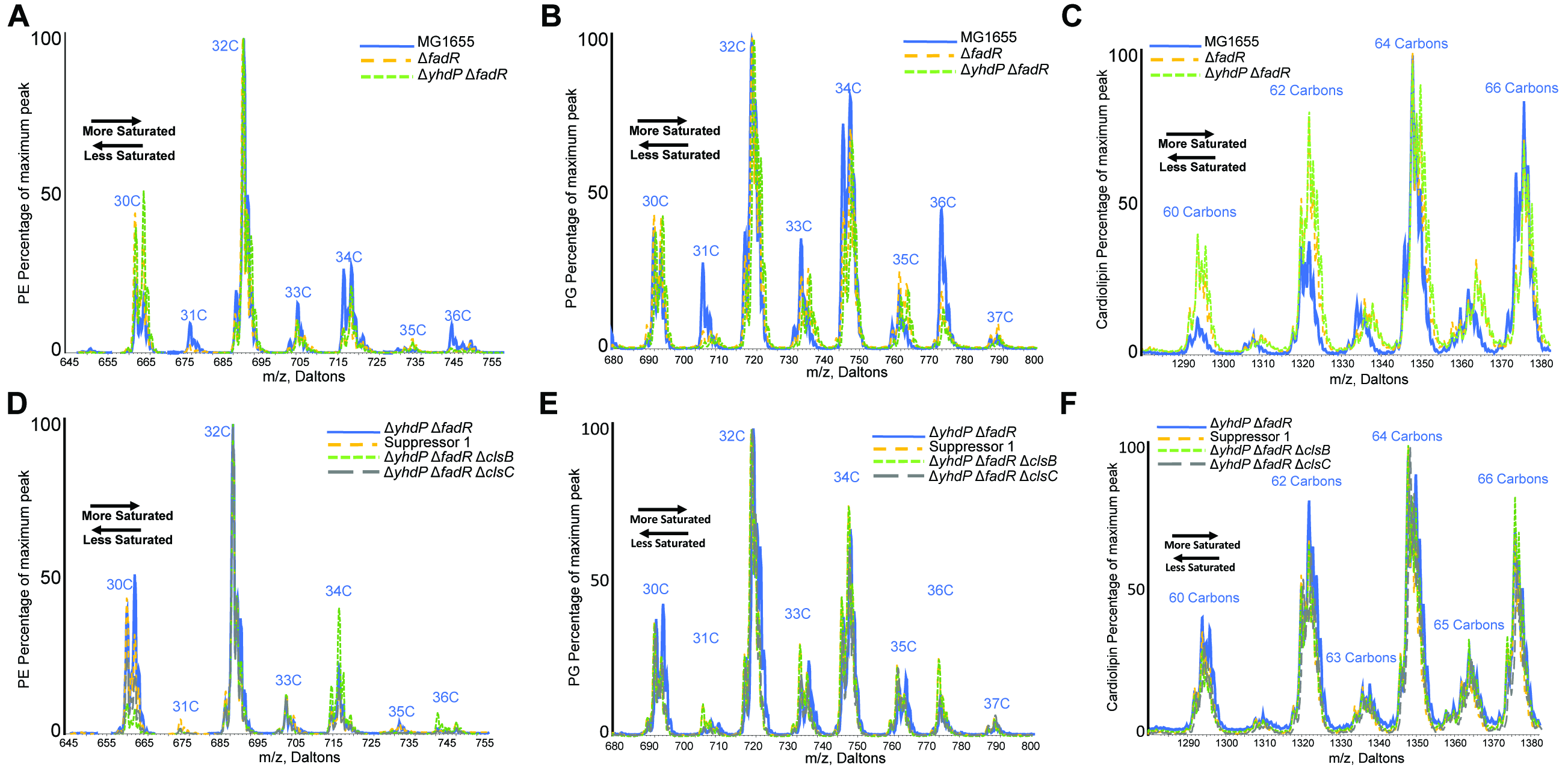

Supplement: S7 Fig — Phospholipid composition of the indicted strains was assayed using LC/MS. Representative spectra are shown the wild type, ΔfadR, and ΔyhdP ΔfadR strains (A-C) and for the ΔyhdP ΔfadR, Suppressor 1, ΔyhdP ΔfadR ΔclsB, and ΔyhdP ΔfadR ΔclsC strains (D-F). Spectra for PE (A, D), PG (B, E), and CL (C, F) are shown as separate panels. Data are shown as relative values to the maximum peak height. (TIF) [file pgen.1011335.s007.tif]

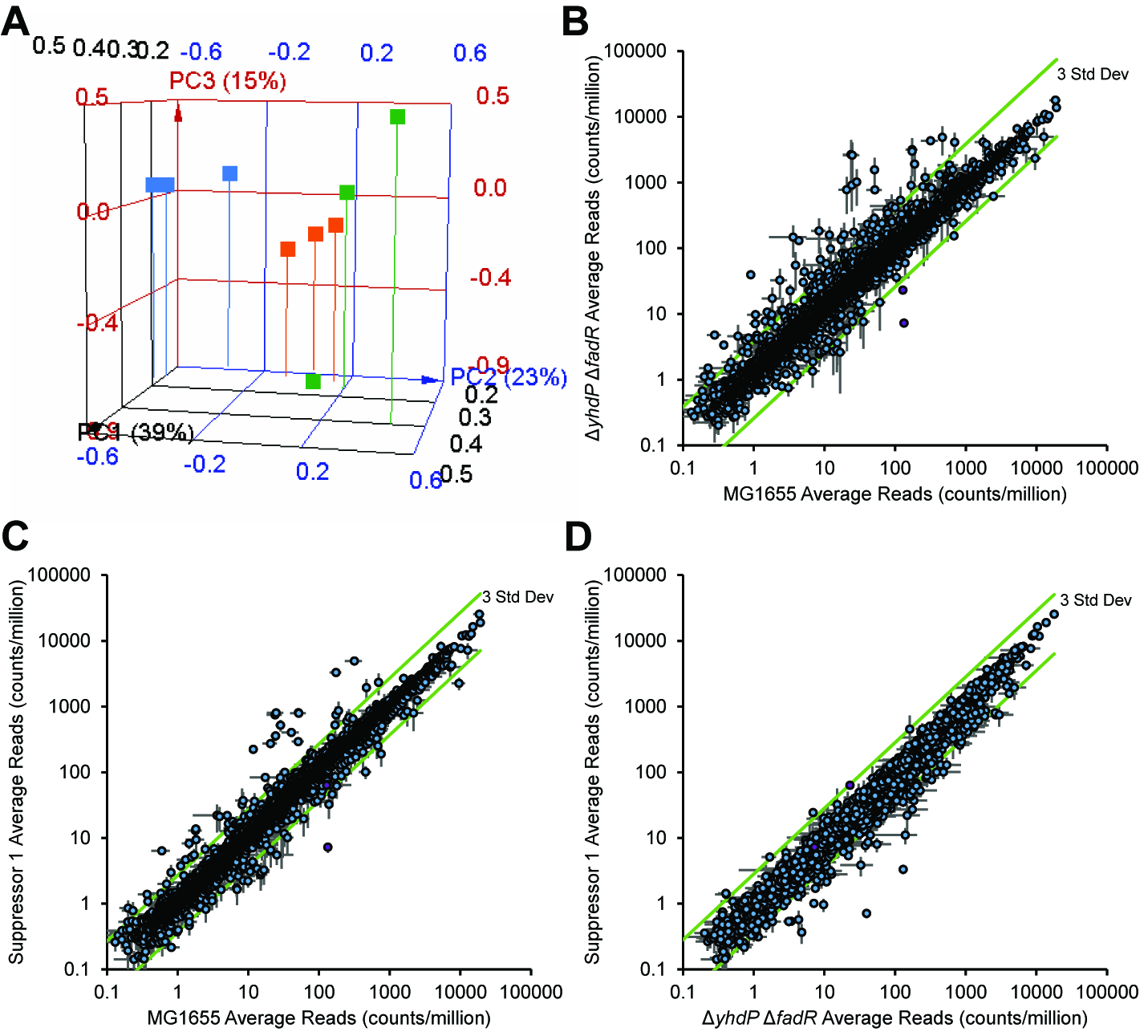

Supplement: S8 Fig — RNA-seq was performed for the wild-type, ΔyhdP ΔfadR, and Suppressor 1 strains (see main text for details). (A) Principal component analysis was performed for the expression of all genes differentially expressed between any two groups. The samples are graphed in relation to the top three principal components. Blue: wild type; Green: ΔyhdP ΔfadR; Orange: Suppressor 1. Suppressor 1 and the ΔyhdP ΔfadR strain cluster most closely together. (B-D) The mean read count per gene ± the SEM is shown for (A) the ΔyhdP ΔfadR strain vs. wild type, (C) Suppressor 1 vs. wild type, and (D) Suppressor 1 vs. the ΔyhdP ΔfadR strain. (TIF) [file pgen.1011335.s008.tif]

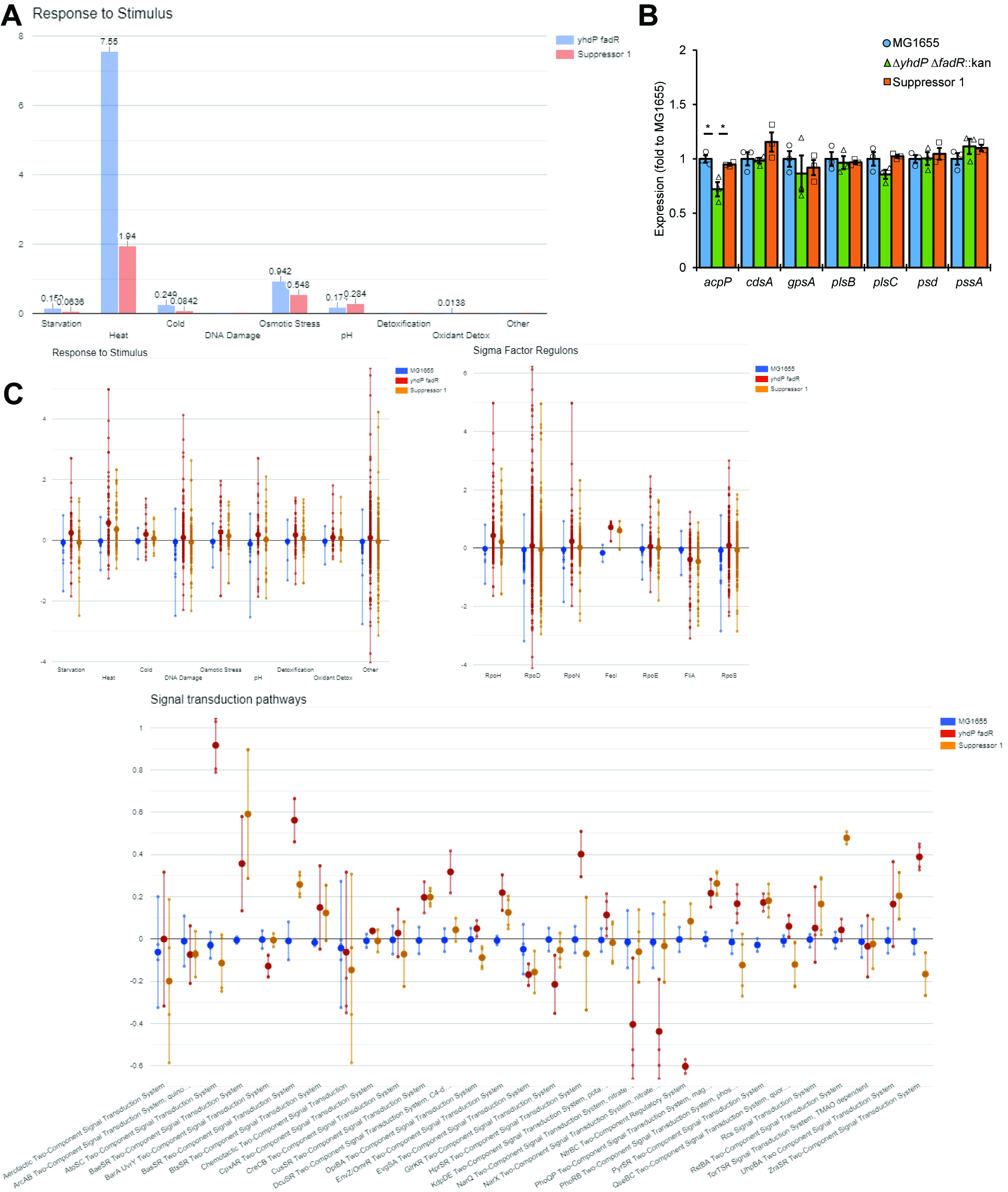

Supplement: S9 Fig — (A) Pathway enrichment analysis was performed on RNA-seq data from the ΔyhdP ΔfadR strain and Suppressor 1 against wild type using the EcoCyc Omics Dashboard. Data for genes in responses to external stimuli are shown. Graphed values at–log(p-values) calculated by a Fisher-exact test. (B) Relative expression of genes in phospholipid biosynthesis not specific for CL synthesis. Data are shown as mean ± SEM and individual data points. * p<0.05 by quasi-linear F-test. (C) Pathway analysis was performed using the EcoCyc Omics Dashboard on the expression of all genes. Data for response to stimulus, sigma factor regulons, and signal transduction pathways are shown as fold values. Large dots indicate the mean for the pathway while small dots indicate individual genes. Lines indicate the range of changes for the pathway. (TIF) [file pgen.1011335.s009.tif]
