# Supplementary material for: Genetic evidence for functional diversification of gram-negative intermembrane phospholipid transporters
Source: PLoS Genet. 2024 Jun 24;20(6):e1011335. doi: 10.1371/journal.pgen.1011335 (PMC11226057; doi:10.1371/journal.pgen.1011335)
Supplement: S1 Table — (DOCX) [file pgen.1011335.s010.docx]

**Table S1: RNA-Seq Descriptive Statistics**

| **Read Alignment Statistics** | | | | | |
| --- | --- | --- | --- | --- | --- |
| **Sample** | **Total Reads** | **All Mapped Reads** | **Coding sequence Mapped Reads** | **rRNA Mapped Reads** | **tRNA Mapped Reads** |
| **MG1655 1** | 35,023,454 | 35,000,224 | 29,647,965 | 27,966 | 92,803 |
| **MG1655 2** | 34,950,112 | 34,920,774 | 28,513,228 | 28,134 | 245,155 |
| **MG1655 3** | 36,311,924 | 36,277,404 | 30,568,531 | 43,327 | 180,315 |
| **∆*yhdP* ∆*fadR* 1** | 36,218,546 | 36,052,383 | 30,202,733 | 28,331 | 164,775 |
| **∆*yhdP* ∆*fadR* 2** | 35,056,248 | 34,914,159 | 27,808,209 | 24,290 | 251,332 |
| **∆*yhdP* ∆*fadR* 3** | 34,920,436 | 34,789,325 | 28,934,121 | 36,209 | 189,229 |
| **Suppressor 1 1** | 33,074,066 | 32,998,982 | 27,683,364 | 27,599 | 120,694 |
| **Suppressor 1 2** | 32,258,252 | 32,189,270 | 26,838,003 | 23,141 | 180,518 |
| **Suppressor 1 3** | 33,379,798 | 33,330,014 | 21,515,004 | 612,947 | 108,837 |
| **Median** | **34,950,112** | **34,914,159** | **28,513,228** | **28,134** | **180,315** |

| **Log2 Fold Values Between Indicated Sample Groups** | | | |
| --- | --- | --- | --- |
| **Statistic** | **∆*yhdP* ∆*fadR*/ MG1655** | **Suppressor 1/ ∆*yhdP* ∆*fadR*** | **Suppressor 1/ MG1655** |
| **Mean** | 0.139 | -0.134 | 0.005 |
| **Median** | 0.055 | -0.052 | -0.012 |
| **Standard Deviation** | 0.650 | 0.499 | 0.477 |
| **Maximum** | 6.80 | 3.61 | 5.02 |
| **Minimum** | -4.20 | -5.78 | -4.21 |
