# Supplementary material for: Genetic evidence for functional diversification of gram-negative intermembrane phospholipid transporters
Source: PLoS Genet. 2024 Jun 24;20(6):e1011335. doi: 10.1371/journal.pgen.1011335 (PMC11226057; doi:10.1371/journal.pgen.1011335)
Supplement: S2 Table — (DOCX) [file pgen.1011335.s011.docx]

**Table S2: RNA-Seq KEGG Pathway Enrichment Analysis**

| **Pathway Identifier** | **Pathway** | **N** | **MG1655 vs ∆*yhdP ∆fadR*** | | | | **∆*yhdP ∆fadR* vs. Suppressor 1** | | | | **MG1655 vs. Suppressor 1** | | | |
| --- | --- | --- | --- | --- | --- | --- | --- | --- | --- | --- | --- | --- | --- | --- |
|  |  |  | **Up** | **Down** | **P. Up** | **P. Down** | **Up** | **Down** | **P. Up** | **P. Down** | **Up** | **Down** | **P. Up** | **P. Down** |
| **path:eco00020** | **Citrate cycle (TCA cycle)** | 29 | 4 | 0 | 0.003 | 1 | 0 | 0 | 1 | 1 | 0 | 0 | 1 | 1 |
| **path:eco00190** | **Oxidative phosphorylation** | 43 | 4 | 0 | 0.012 | 1 | 0 | 0 | 1 | 1 | 0 | 0 | 1 | 1 |
| **path:eco00410** | **beta-Alanine metabolism** | 14 | 2 | 0 | 0.033 | 1 | 0 | 0 | 1 | 1 | 1 | 0 | 0.05571 | 1 |
| **path:eco00620** | **Pyruvate metabolism** | 59 | 5 | 0 | 0.007 | 1 | 0 | 0 | 1 | 1 | 1 | 0 | 0.215643 | 1 |
| **path:eco00650** | **Butanoate metabolism** | 35 | 7 | 0 | 5.68E-06 | 1 | 0 | 1 | 1 | 0.148376 | 1 | 0 | 0.133825 | 1 |
| **path:eco01220** | **Degradation of aromatic compounds** | 17 | 2 | 0 | 0.048 | 1 | 0 | 1 | 1 | 0.074888 | 0 | 0 | 1 | 1 |
| **path:eco02026** | **Biofilm formation - Escherichia coli** | 52 | 4 | 0 | 0.022 | 1 | 0 | 0 | 1 | 1 | 0 | 0 | 1 | 1 |
| **path:eco00627** | **Aminobenzoate degradation** | 6 | 1 | 0 | 0.118978 | 1 | 0 | 1 | 1 | 0.027 | 0 | 0 | 1 | 1 |
| **path:eco00630** | **Glyoxylate and dicarboxylate metabolism** | 42 | 2 | 0 | 0.218309 | 1 | 0 | 0 | 1 | 1 | 2 | 0 | 0.012 | 1 |
| **path:eco01212** | **Fatty acid metabolism** | 21 | 5 | 1 | 5.53E-05 | 0.073103 | 0 | 0 | 1 | 1 | 3 | 1 | 7.17E-05 | 0.020 |
| **path:eco00061** | **Fatty acid biosynthesis** | 13 | 0 | 1 | 1 | 0.046 | 0 | 0 | 1 | 1 | 0 | 1 | 1 | 0.012 |
| **path:eco00071** | **Fatty acid degradation** | 15 | 5 | 0 | 9.00E-06 | 1 | 0 | 0 | 1 | 1 | 3 | 0 | 2.49E-05 | 1 |
| **path:eco00280** | **Valine, leucine and isoleucine degradation** | 11 | 4 | 0 | 5.23E-05 | 1 | 0 | 0 | 1 | 1 | 2 | 0 | 8.43E-04 | 1 |
| **path:eco00281** | **Geraniol degradation** | 6 | 4 | 0 | 2.58E-06 | 1 | 0 | 0 | 1 | 1 | 2 | 0 | 2.33E-04 | 1 |
| **path:eco00362** | **Benzoate degradation** | 12 | 5 | 0 | 2.50E-06 | 1 | 0 | 0 | 1 | 1 | 2 | 0 | 0.001 | 1 |
| **path:eco00380** | **Tryptophan metabolism** | 10 | 2 | 0 | 0.017 | 1 | 0 | 0 | 1 | 1 | 1 | 0 | 0.040 | 1 |
| **path:eco00592** | **alpha-Linolenic acid metabolism** | 3 | 2 | 0 | 0.001 | 1 | 0 | 0 | 1 | 1 | 1 | 0 | 0.012 | 1 |
| **path:eco00903** | **Limonene and pinene degradation** | 3 | 2 | 0 | 0.001 | 1 | 0 | 0 | 1 | 1 | 1 | 0 | 0.012 | 1 |
| **path:eco00930** | **Caprolactam degradation** | 3 | 2 | 0 | 0.001 | 1 | 0 | 0 | 1 | 1 | 1 | 0 | 0.012 | 1 |
| **path:eco01110** | **Biosynthesis of secondary metabolites** | 338 | 12 | 0 | 0.047 | 1 | 0 | 0 | 1 | 1 | 4 | 0 | 0.043 | 1 |
| **path:eco01120** | **Microbial metabolism in diverse environments** | 268 | 14 | 0 | 0.001 | 1 | 0 | 2 | 1 | 0.348026 | 4 | 0 | 0.020 | 1 |
| **path:eco01200** | **Carbon metabolism** | 110 | 8 | 0 | 0.002 | 1 | 0 | 0 | 1 | 1 | 3 | 0 | 0.009 | 1 |
