# Supplementary material for: Genetic evidence for functional diversification of gram-negative intermembrane phospholipid transporters
Source: PLoS Genet. 2024 Jun 24;20(6):e1011335. doi: 10.1371/journal.pgen.1011335 (PMC11226057; doi:10.1371/journal.pgen.1011335)
Supplement: S3 Table — (DOCX) [file pgen.1011335.s012.docx]

**Table S3: Strains used in this study**

| **Strain** | **Genotype** | **Reference** |
| --- | --- | --- |
| MG1655 | K-12 F^-^ λ^-^ *rph-1* | (1) |
| AM182 | MG1655 Δ*yhdP* | (2) |
| AK38 | MG1655 Δ*fadR*::cm | This study |
| AK31 | MG1655 Δ*fadR*::kan | This study |
| AK7 | MG1655 Δ*yhdp* Δ*fadR*::kan | This study |
| AK58 | MG1655 Δ*yhdp* Δ*fadR*::cm | This study |
| AM174 | MG1655 Δ*wecA*::kan | (3) |
| AM334 | MG1655 Δ*wecA* | (2) |
| AM365 | MG1655 Δ*wzzE* | (2) |
| AK183 | MG1655 Δ*yhdp* Δ*fadR*::cm Δ*wecA*::kan | This study |
| AM1442 | MG1655 Δ*tamB*::kan | This study |
| AK176 | MG1655 Δ*ydbH*::kan | This study |
| AM341 | MG1655 Δ*yhdP* Δ*wecA* | (2) |
| AM369 | MG1655 Δ*yhdP* Δ*wzzE* | (2) |
| AM1444 | MG1655 Δ*wecA* Δ*tamB*::kan | This study |
| AM1445 | MG1655 Δ*wecA* Δ*ydbH*::kan | This study |
| AM1446 | MG1655 Δ*wzzE* Δ*tamB*::kan | This study |
| AM1447 | MG1655 Δ*wzzE* Δ*ydbH*::kan | This study |
| AK113 | MG1655 Δ*tamB*::kan Δ*fadR*::cm | This study |
| AK177 | MG1655 Δ*ydbH*::kan Δ*fadR*::cm | This study |
| AM743 | MG1655 Δ*elyC* | (4) |
| AK3 | MG1655 Δ*yhdp* Δ*fadR*::kan P*_fabA_*(-19G>A) (Suppressor 1) | This study |
| AK200 | MG1655 Δ*yhdp* Δ*fadR*::kan *zcb*-3059::Tn*10* | This study; Tn*10* allele from (5) |
| AK201 | MG1655 Δ*yhdp* Δ*fadR*::kan *zcb*-3059::Tn*10* P*_fabA_*(-19G>A) | This study |
| AM384 | MG1655 Δ*pldA*::kan | This study |
| AM385 | MG1655 Δ*yhdp* Δ*pldA*::kan | This study |
| AM522 | MG1655 Δ*mlaA*::kan | (2) |
| AM524 | MG1655 Δ*yhdp* Δ*mlaA*::kan | This study |
| AK182 | MG1655 Δ*yhdp* Δ*fadR*::cm Δ*pldA*::kan | This study |
| AK180 | MG1655 Δ*yhdp* Δ*fadR*::cm Δ*mla*::kan | This study |
| AK167 | MG1655 Δ*clsA*::kan | This study |
| AK168 | MG1655 Δ*clsB*::kan | This study |
| AK169 | MG1655 Δ*clsC*::kan | This study |
| AM1357 | MG1655 Δ*clsA* Δ*clsB* Δ*clsC* | This study |
| AK171 | MG1655 Δ*yhdP* Δ*fadR*::cm Δ*clsA*::kan | This study |
| AK172 | MG1655 Δ*yhdP* Δ*fadR*::cm Δ*clsB*::kan | This study |
| AK173 | MG1655 Δ*yhdP* Δ*fadR*::cm Δ*clsC*::kan | This study |
| AK274 | MG1655 Δ*clsA* Δ*clsB* Δ*clsC* Δ*yhdP* Δ*fadR*::cm | This study |
| AM1409 | MG1655 Δ*rcsF* Δ*lpp* Δ*pgsA* pBAD33-pgsA | (6) |
| AK205 | MG1655 Δ*rcsF* Δ*lpp* Δ*pgsA* Δ*yhdP*::kan *fadR*::Tn*10* pBAD33-pgsA | This study |
| AK89 | MG1655 Δ*fabR*::kan | This study |
| AK84 | MG1655 Δ*yhdP* Δ*fadR*::cm Δ *fabR*::kan | This study |
| AK178 | MG1655 Δ*yhdP* Δ*fadR*::cm Δ*tamB*::kan | This study |
| AM1469 | MG1655 Δ*yhdP* Δ*tamB*::kan | This study |
| AK179 | MG1655 Δ*yhdP* Δ*fadR*::cm Δ*ydbH*::kan | This study |
| AM944 | MG1655 pJW15 | (4); plasmid from (7) |
| AK140 | MG1655 pJW15-P*_fabA_* | This study |
| AK141 | MG1655 pJW15-P*_fabA_*(-19G>A) | This study |
| AK142 | MG1655 Δ*yhdP* pJW15-P*_fabA_* | This study |
| AK143 | MG1655 Δ*yhdP* pJW15-P*_fabA_*(-19G>A) | This study |
| AK144 | MG1655 Δ*fadR*::cm pJW15-P*_fabA_* | This study |
| AK145 | MG1655 Δ*fadR*::cm pJW15-P*_fabA_*(-19G>A) | This study |
| AK146 | MG1655 Δ*yhdP* Δ*fadR*::cm pJW15-P*_fabA_* | This study |
| AK147 | MG1655 Δ*yhdP* Δ*fadR*::cm pJW15-P*_fabA_*(-19G>A) | This study |
| AK148 | MG1655 Δ*fabR* pJW15-P*_fabA_* | This study |
| AK149 | MG1655 Δ*fabR* pJW15-P*_fabA_*(-19G>A) | This study |

**References**

1. Guyer MS, Reed RR, Steitz JA, Low KB. Identification of a sex-factor-affinity site in E. coli as gamma delta. Cold Spring Harb Symp Quant Biol. 1981;45 Pt 1:135-40.

2. Mitchell AM, Srikumar T, Silhavy TJ. Cyclic enterobacterial common antigen maintains the outer membrane permeability barrier of *Escherichia coli* in a manner controlled by Yhdp. mBio. 2018;9(4):e01321-18.

3. Mitchell AM, Wang W, Silhavy TJ. Novel Rpos-dependent mechanisms strengthen the envelope permeability barrier during stationary phase. J Bacteriol. 2017;199(2):e00708-16.

4. Rai AK, Carr JF, Bautista DE, Wang W, Mitchell AM. ElyC and cyclic enterobacterial common antigen regulate synthesis of phosphoglyceride-linked enterobacterial common antigen. mBio. 2021;12(6):e02846-21.

5. Singer M, Baker TA, Schnitzler G, Deischel SM, Goel M, Dove W, et al. A collection of strains containing genetically linked alternating antibiotic resistance elements for genetic mapping of Escherichia coli. Microbiol Rev. 1989;53(1):1-24.

6. Morris KN, Mitchell AM. Phosphatidylglycerol Is the Lipid Donor for Synthesis of Phospholipid-Linked Enterobacterial Common Antigen. Journal of Bacteriology. 2023;205(1):e00403-22.

7. MacRitchie DM, Ward JD, Nevesinjac AZ, Raivio TL. Activation of the Cpx envelope stress response down-regulates expression of several locus of enterocyte effacement-encoded genes in enteropathogenic *Escherichia coli*. Infect Immun. 2008;76(4):1465-75.
