# Supplementary material for: Genetic evidence for functional diversification of gram-negative intermembrane phospholipid transporters
Source: PLoS Genet. 2024 Jun 24;20(6):e1011335. doi: 10.1371/journal.pgen.1011335 (PMC11226057; doi:10.1371/journal.pgen.1011335)
Supplement: S4 Table — (DOCX) [file pgen.1011335.s013.docx]

**Table S4. Primers used in this study**

| **Primer** | **Sequence (5ʹ to 3ʹ)** |
| --- | --- |
| K-*fadR*-FP (*fadR*::cm recombineering) | TCTGGTATGATGAGTCCAACTTTGTTTTGCTGTGTTATGGAAATCTCACTATGTGTAGGCTGGAGCTGCTTCG |
| K-*fadR*-RP (*fadR*::cm recombineering) | AACAACAAAAAACCCCTCGTTTGAGGGGTTTGCTCTTTAAACGGAAGGGACATATGAATATCCTCCTTAG |
| Overlap-pJW15-*fabA* FP | GGCCCTTTCGTCTTCACCTCGGAATCAGAGTATCGCTATCACAG |
| Overlap-pJW15-*fabA* RP | GATCCGGTACCCGGGCTGCAGTTCTCTGTAAGCCTTATTTTATTG |
| Overlap-pJW15-*fabA* FP1 | TGCAGCCCGGGTACCGGATCCTCTAGTTGCGGCCGCAAAATG |
| Overlap-pJW15-*fabA* RP1 | CGAGGTGAAGACGAAAGGGCCTCGTGATACGCCTATTTTTATAGG |
